# Diurnal T2-changes of the intervertebral discs of the entire spine and the influence of weightlifting

**DOI:** 10.1038/s41598-020-71003-z

**Published:** 2020-09-01

**Authors:** Thorsten Jentzsch, Nadja A. Farshad-Amacker, Philipp Mächler, Jan Farei-Campagna, Armando Hoch, Andrea B. Rosskopf, Clément M. L. Werner

**Affiliations:** 1 Department of Trauma, University Hospital Zurich, University of Zurich, Zurich, Switzerland; 2 Department of Orthopaedics, Balgrist University Hospital, Zurich, Switzerland; 3 Department of Radiology, University Hospital Zurich, Zurich, Switzerland; 4 Department of Radiology, Balgrist University Hospital, Zurich, Switzerland

**Keywords:** Cartilage, Risk factors

## Abstract

The purpose was to study if (1) diurnal changes occur in the entire spine and if (2) intervertebral discs (IVDs) of weightlifters (WL) have decreased baseline T2-values in the morning as well as (3) increased diurnal changes throughout the day. This prospective cohort study investigated healthy volunteers between 2015 and 2017. WL were required to have participated in weightlifting ≥ 4×/week for ≥ 5 years, while non-weightlifters (NWL) were limited to < 2×/week for ≥ 5 years. Both groups underwent magnetic resonance imaging (MRI) of the entire spine in the morning and evening. WL were requested to perform weightlifting in-between imaging. IVD regions of interest (nucleus pulposus) were defined and T2-maps were measured. Analysis consisted of unpaired t-test, paired t-test, propensity-score matching (adjusting for age and sex), and Pearson correlation. Twenty-five individuals (15 [60.0%] males) with a mean age of 29.6 (standard deviation [SD 6.9]) years were analyzed. Both groups (WL: n = 12 versus [vs.] NWL: n = 13) did not differ demographic characteristics. Mean IVD T2-values of all participants significantly decreased throughout the day (95.7 [SD 15.7] vs. 86.4 [SD 13.9] milliseconds [ms]) in IVDs of the cervical (71.8 [SD 13.4] vs. 64.4 [SD 14.1] ms), thoracic (98.8 [SD 19.9] vs. 88.6 [SD 16.3] ms), and lumbar (117.0 [SD 23.7] vs. 107.5 [SD 21.6] ms) spine (P < 0.001 each). There were no differences between both groups in the morning (P = 0.635) and throughout the day (P = 0.681), even after adjusting for confounders. It can be concluded that diurnal changes of the IVDs occurred in the entire (including cervical and thoracic) spine. WL and NWL showed similar morning baseline T2-values and diurnal changes. Weightlifting may not negatively affect IVDs chronically or acutely.

## Introduction

The intervertebral discs (IVDs) of the spine are responsible for about 25 percent (%) of the spinal height. Their diurnal changes may decrease spinal height up to almost 2 cm or about 1% of total body height throughout the day, while recovering during the night^1,2^. These changes have been attributed to increased hydrostatic pressure compared to osmotic pressure in an upright posture. This specifically affects the nucleus pulposus due to its high water (around 70%) and proteoglycan content compared to the annulus fibrosus. Degenerated IVDs lose their ability to completely recover from daily compressive forces due to limited capacity of water reabsorption^3^.

Newer imaging modalities may help in characterizing diurnal changes in the entire spine and in clarifying the association of weightlifting and IVDs. T2-mapping is a magnetic resonance imaging (MRI) technology that visualizes water^4^ and proteoglycan content better allowing earlier quantitative evaluation of cartilage change and degeneration than conventional T1- and T2-weighted images^5,6^. Regional T2-values (or transverse magnetization relaxation times as observed in multi-echo spin-echo experiments) are calculated by mono-exponential curve fitting of the signal-intensity decay as a function of echo time in all voxels of an imaging series and can be summarized and visually inspected in the form of quantitative T2-maps^7^. T2-mapping has been described in several studies at the extremities of the body, such as the knee^8^, and in the evaluation of IVD degeneration^9–11^, but a recent study by Chokan et al.^12^ was the first to use in vivo T2-mapping in order to quantify water content of the IVD^4^ in the lumbar spine before and after exercise. It was shown that T2-values decreased in the nucleus pulposus after exercise and increased again after rest. This was confirmed in another study by Yamabe et al.^13^. Therefore, it was concluded that T2-mapping was useful in evaluating IVD function. There remains conflicting evidence about the influence of weightlifting on IVDs with some evidence that weightlifting affects degeneration of the IVDs in some parts of the spine^14^, but some other evidence stating the contrary^15,16^. It remains unknown if diurnal changes can be shown with T2-mapping, whether they are also present in the cervical and thoracic spine, and if weightlifting influences these changes chronically and/or acutely.

### Main aim

The study hypotheses were that (1) diurnal changes occur in the entire spine as well as (2) IVDs of weightlifters (WL) have decreased baseline T2-mapping values (possibly water content) in the morning and (3) increased diurnal changes throughout the day.

## Materials and methods

### Participants

Two groups, WL and non-weightlifters (NWL), were recruited. The inclusion criteria for both groups were as follows: WL were required to have participated in weightlifting ≥ 4 times per week for ≥ 5 years, while NWL were limited to a maximum participation in weightlifting of < 2 times per week for ≥ 5 years. Exclusion criteria were age < 18 years, any disease of the spine, symptoms at the spine, and contraindications for MRI.

### Procedures

Both groups, WL and NWL, underwent MRI of the entire spine in the morning (around 7 a.m.) after bedrest and evening (around 4 p.m.). WL were requested to perform a heavy weightlifting exercise of the entire body with specific exercises in-between imaging, while NWL were asked to refrain from any heavy activity. The time period between the workout and MRI in the evening was ≥ 30 min^12^.

### Magnetic resonance imaging

A supine MRI of the entire (cervical, thoracic, and lumbar) spine was acquired on a 3 Tesla scanner (Skyra; Siemens, Erlangen, Germany) using a body coil. The imaging protocol included sagittal T2-weighted fast-spin-echo and sagittal multi-echo spin-echo sequences for quantitative T2-mapping. Image parameters were as follows: repetition time 2,000 ms (ms), echo time 12 ms, slice thickness 4 mm (mm), intersection gap 0.8 mm, number of excitations 10, echo train length 6, and matrix size 384 × 384.

All image interpretations and measurements were performed using the program OsirisX^17^. Quantitative measurements were applied in the sagittal plane using T2-mapped images by a resident, who had been specifically trained by an attending physician. A rectangular region of interest (ROI) of 2.3 mm (horizontal) and 1.3 mm (vertical) side length was placed in the center of each intervertebral disc (nucleus pulposus) on sagittal T2-maps obtained in the morning and evening. The horizontal and vertical positions of each ROI within each IVD was recorded and used to position the ROI with identical side lengths in the same locations for each (morning and evening) acquisition. Further, the Pfirrmann classification for IVD degeneration was used to qualitatively grade the degree of degeneration in each disc in the morning as well as the evening images^18^.

### Weightlifting

Weightlifting in-between MRIs consisted of a heavy workout of the entire body with a focus on exercises that stain the spine. It included three sets with 8–12 repetitions (choosing a weight that lead to complete fatigue after a maximum of 12 repetitions) of the following exercises: squats, deadlifts, shoulder front press, bench press, triceps rope pulldowns, and sit-ups.

### Statistical methods

Categorical values are presented as absolute numbers (%). Continuous values are given as means (standard deviation [SD]). The chi-squared, unpaired t-test, paired t-test, propensity-score matching (adjusting for age and sex), and Pearson correlation were used. A post hoc power calculation showed that the power was excellent (power = 1.0 based on alpha = 0.05, n = 25, mean in the morning = 95.7, mean in the evening = 86.4, standard deviation (SD) of the difference = 8.7 for paired samples). The significance level was set at P = 0.05. Stata (version 13.1; StataCorp LLC, College Station, Texas, United States of America) was used.

### Study design, setting, and ethics

This was a prospective cohort study of healthy volunteers conducted between 2015 and 2017. It was approved by the local ethics committee (cantonal ethics committee Zurich (“Kantonale Ethikkommission Zürich”, KEK-ZH-Nr. 2014-0579). All participants gave their informed consent. All methods were performed in accordance with the relevant guidelines and regulations.

## Results

### Participants

Twenty-five individuals were analyzed (WL: n = 12 versus [vs.] NWL: n = 13). The mean age was 29.6 (SD 6.9) years and there were 15 [60.0%] males. There were no differences between groups (age: WL 31.4 [SD 8.7] years vs. NWL 27.9 [SD 4.3] years, P = 0.212; males: WL n = 8 [66.7%] vs. NWL n = 7 [53.8%], P = 0.513).

### Diurnal changes

**Figure 1:**
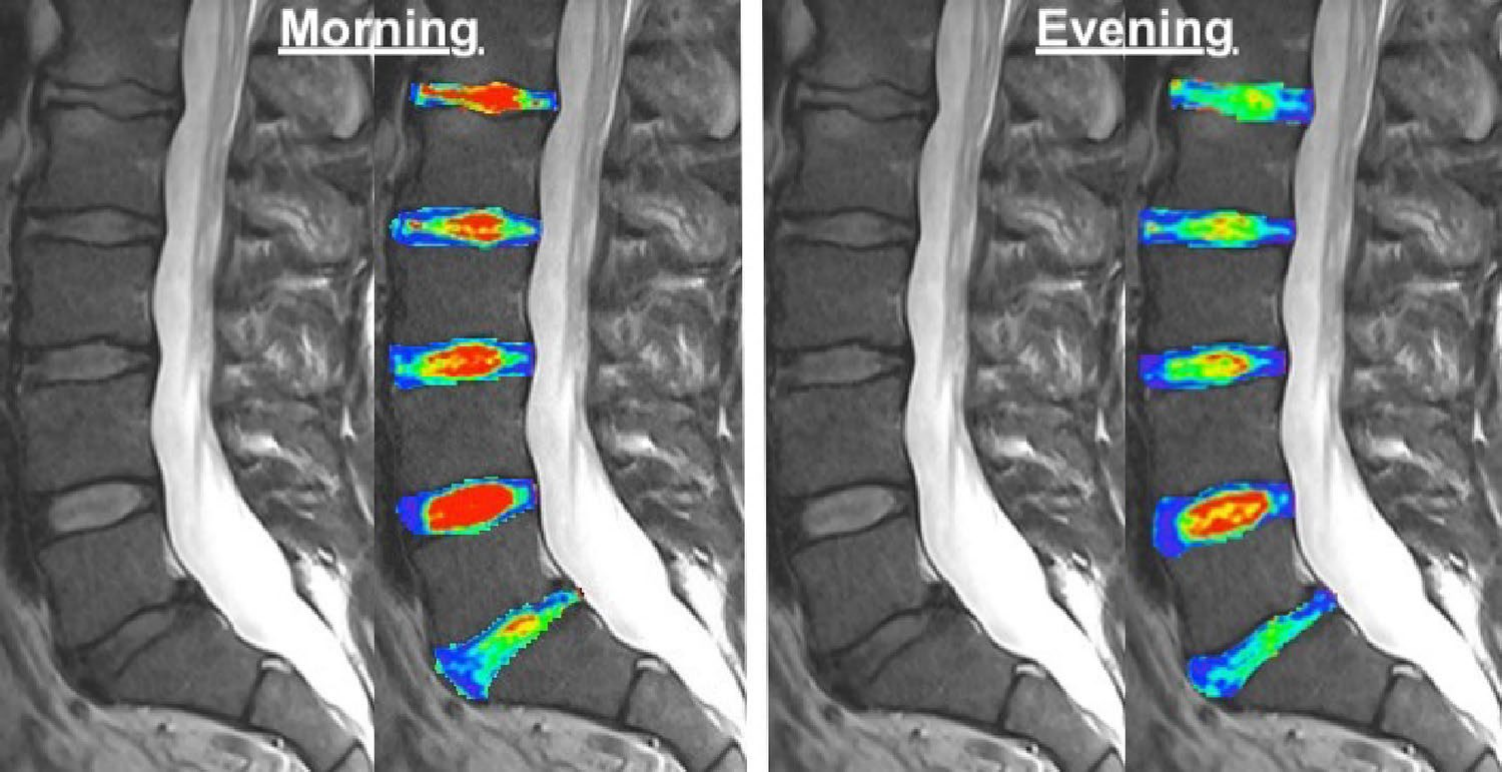
Intervertebral discs (IVDs). These sagittal magnetic resonance images (MRI) of a 53 year old male display higher T2-values of IVDs in the morning (red) than in the evening (green).

**Figure 2:**
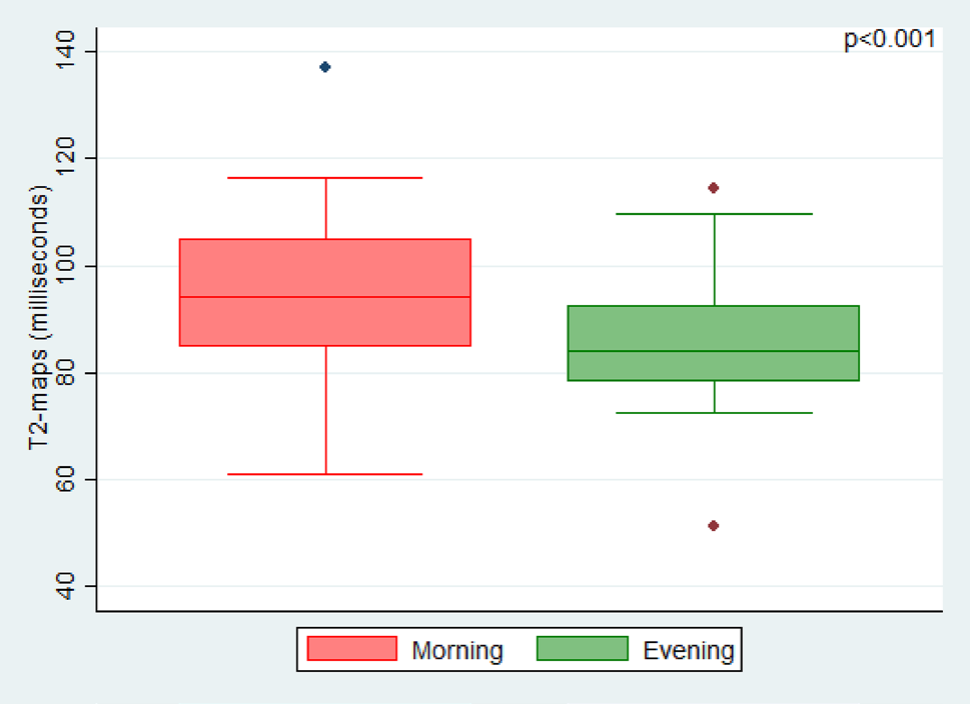
T2-values. This boxplot of the study population (n = 25) shows significantly higher T2-values of intervertebral discs (IVDs) in magnetic resonance images (MRI) in the morning (red) than in the evening (green).

The mean of T2-values within the IVDs significantly decreased throughout the day (morning: 95.7 [SD 15.7] vs. evening: 86.4 [SD 13.9] milliseconds [ms], P < 0.001) (Figs. 1, 2, Table 1). The decrease of T2-values in the IVDs was found in the cervical spine (71.8 [SD 13.4] vs. 64.4 [SD 14.1] ms, P < 0.001), thoracic (98.8 [SD 19.9] vs. 88.6 [SD 16.3] ms, P < 0.001), and lumbar (117.0 [SD 23.7] vs. 107.5 [SD 21.6] ms, P < 0.001) spine. It was noted that the mean T2-mapping values were highest in the lumbar spine and that the decrease in T2-values was highest in the thoracic spine. Overall, there were no changes in the Pfirrmann grades throughout the day (morning mean: 2.1 [SD 0.3] vs. evening mean: 2.1 [SD 0.3], P = 0.180) with very strong correlations in the morning and evening (r = 0.995, P < 0.001).

**Table 1:** Diurnal changes of intervertebral discs (IVDs) (n = 25).

Variable	Morning (n = 25)	Evening (n = 25)	Difference*	P-value^†^
	Mean (SD)			
**T2-values (ms) of spine**				
Entire	95.7 (15.7)	86.4 (13.9)	− 9.3 (8.7)	< 0.001
Cervical	71.8 (13.4)	64.4 (14.1)	− 7.4 (8.3)	< 0.001
Thoracic	98.8 (19.9)	88.6 (16.3)	− 10.1 (11.5)	< 0.001
Lumbar	117.0 (23.7)	107.5 (21.6)	− 9.5 (12.4)	< 0.001

*n* number, *SD* standard deviation, *ms* milliseconds.

*Between morning and evening.

^†^Paired t-test.

### Weightlifting

The IVD T2 values did not significantly differ between both groups in the baseline values in the morning (WL 94.1 [SD 14.1] vs. NWL 97.2 [SD 17.4], P = 0.635), evening (WL 85.6 [SD 14.9] vs. NWL 87.2 [SD 13.5], P = 0.785), and throughout the day (difference between morning and evening: WL − 8.5 [SD 8.9] vs. NWL − 10.0 [SD 8.9], P = 0.681) (Table 2). These non-significant findings in diurnal changes remained unchanged in propensity-score matching analysis adjusting for age and gender differences (P = 0.360).

**Table 2:** Diurnal changes of intervertebral discs (IVDs) according to weightlifting (n = 25).

Variable	WL (n = 12)	NWL (n = 13)	P-value*
	Mean (SD)		
Age (yrs)	31.4 (8.7)	27.9 (4.3)	0.212
Males (%)	8 (66.7)	7 (53.8)	0.513
**T2 values (ms) of spine**			
Morning	94.1 (14.1)	97.2 (17.4)	0.635
Evening	85.6 (14.9)	87.2 (13.5)	0.785
Difference between morning and evening	− 8.5 (8.9)	− 10.0 (8.9)	0.681^†^

*n* number, *SD* standard deviation, *yrs* years, *%* percent, *ms* milliseconds, *vs* versus.

*Unpaired t-test and chi-squared test.

^†^Remained non-significant with propensity-score matching analysis (P = 0.360).

## Discussion

In this relatively young patient cohort, T2 measurements detected significant diurnal changes in IVD nuclei pulposi with substantially lower T2-values in the evening compared to the morning, while Pfirrmann grades remained unchanged. As another novel finding, these results were present in the entire spine, not only affecting the lumbar, but also the cervical and thoracic spine. We were also able to show that the lumbar spine showed higher mean T2-mapping values than the thoracic and cervical spine. The baseline levels and, surprisingly, also the diurnal changes were similar in WL and in NWL. Thus, it is possible that the water content of the IVDs may not be influenced by heavy weightlifting; neither chronically in the baseline levels, nor acutely in the diurnal changes. In other words, long-standing weightlifting did not seem to be associated with differences in hydration of IVDs after bedrest and T2 changes after a heavy weightlifting workout. This in turn may be interpreted as no differences in degeneration of the IVDs, as Watanabe et al. have described decreased T2-values in degenerated IVDs^6^ and rehydration capacity^4^.

There is conflicting evidence about the influence of weightlifting on IVDs with some evidence that weightlifting affects degeneration of the IVDs, but some other evidence stating the contrary. A previous study from Videman et al.^14^ about 22 and 12 discordant monozygotic twin pairs, demonstrated that endurance exercise did not affect degeneration of the IVDs, while weightlifting had mild effects only on the thoracic, but not the lumbar spine. This is contrasted by reports, in which only little (around 10%) of the variability in degeneration was explained by weightlifting; for example, when comparing former elite weightlifters to shooters after participating a mean of 26 years in their respective sport^15,16^. Antoniou et al. correlated MRI parameters with biochemical analysis^4^. They were able to show that T1- and T2 values in the nucleus pulposus is correlated with the matrix and water integrity of an IVD. A study from Ellingson et al. investigated residual stress and strain on 18 human cadaveric lumbar IVDs with T2*-mapping and showed that significant correlations between radiological and histological water changes in the IVDs^11^. So far, only Chokan et al. and Yamabe et al. have studied the in-vivo effect of exercise on the IVDs^12^ as well as IVDs and facet joints^13^ using T2-mapping. In the former study, an MRI was conducted in 40 individuals three times; before exercise, immediately after exercise, and after 30 min rest. Exercises consisted of 15 repetitions of 30° extension, 45° flexion, and 40° rotation. It was nicely shown that T2-values in the nucleus pulposus of the IVDs decreased immediately after exercise and increased again after rest, with no significant differences between the values before exercise and after rest. Overall, they provided evidence that the nucleus pulposus functioned as a shock absorber due to its water retention with virtually complete regeneration within 30 min after strain. However, they called for an evaluation of diurnal changes in the IVDs using T2-mapping, which we have gladly undertaken with our study. They also limited their investigation to the lumbar spine. Our study may have used a heavier workout program and confirms that exercise decreases T2-values in the lumbar spine, adds that these findings are also observed as part of diurnal changes and that they are also present in the cervical and thoracic spine. However, we did not find differences in WL and NWL chronically or acutely. Future studies may opt to perform an MRI immediately after weightlifting.

Our study found the highest mean T2-mapping-values in the lumbar spine, followed by the thoracic and cervical spine. This is somewhat different to a previous study by Belavy et al.^19^, who reported higher T2-weighted values for the thoracic than the lumbar and cervical spine. The reason for these differences may be found in different imaging protocols and ROIs. However, the fact that IVDs and, thus, also the nucleus pulposus of the lumbar spine are larger might support our finding, particularly in this young study cohort with less degenerated IVDs.

Certain limitations need consideration. First, the volunteer cohort was rather young limiting the generalizability. We focused specifically on young individuals who have only little disc degeneration. It has been previously shown, that T2 mapping can be particularly used in early diagnosis of disc degeneration^20,21^. In older individuals, who have various disc degeneration degrees, certain findings could potentially be masked. Future studies may focus on elderly individuals since findings may be different with a longer life-time exposure to weightlifting. Second, the sample size was rather small, but recruiting participants for such a study with strict inclusion criteria and time-consuming MRI imaging of the entire spine twice a day as well as performing a heavy workout in-between imaging is resource-intense. Furthermore, the sample size analysis revealed a sufficient power. Third, our inclusion criteria allowed the inclusion of hobby weightlifters. So, professional weightlifters may show different results. In theory, however, weightlifting ≥ 4 times per week for ≥ 5 years is sufficient to have put enough strain on the spine in order to warrant investigation of differences between our chosen groups. Future studies may also choose to study differences in these subgroups of amateurs and professionals. Fourth, bias could have been introduced by IVD loss throughout the day, which may in turn could have changed the relative size of the disc height to the ROI. We opted not to change the size of the ROI to avoid introduction of an even more problematic bias. To further address this issue, it was made sure that the size of the ROI was identical in the morning and evening acquisitions. Furthermore, the maximum IVD T2-values showed similar findings (i.e. decreases throughout the day [112.2 (SD 20.2) vs. 99.9 (SD 17.4) ms; P < 0.001]) to the mean IVD T2 values indicating that the risk of bias is extremely low. Fifth, it was not possible to quantify the exact strain on the lumbar spine during the weightlifting workout. However, each individual was instructed to train as intense as possible and exercises of a regular workout routine were chosen.

## Conclusion

In this relatively young, healthy cohort population, diurnal changes of the IVDs occurred in the entire (including cervical and thoracic) spine, possibly due to reduced water content. WL and NWL showed similar baseline morning T2 values and diurnal changes. Therefore, (hobby) weightlifting may not negatively affect IVDs chronically or acutely.
